# Deficiency of autism risk factor *ASH1L* in prefrontal cortex induces epigenetic aberrations and seizures

**DOI:** 10.1038/s41467-021-26972-8

**Published:** 2021-11-15

**Authors:** Luye Qin, Jamal B. Williams, Tao Tan, Tiaotiao Liu, Qing Cao, Kaijie Ma, Zhen Yan

**Affiliations:** Department of Physiology and Biophysics, State University of New York at Buffalo, Jacobs School of Medicine and Biomedical Sciences, Buffalo, NY 14203 USA

**Keywords:** Autism spectrum disorders, Epilepsy, Cellular neuroscience

## Abstract

*ASH1L*, a histone methyltransferase, is identified as a top-ranking risk factor for autism spectrum disorder (ASD), however, little is known about the biological mechanisms underlying the link of *ASH1L* haploinsufficiency to ASD. Here we show that ASH1L expression and H3K4me3 level are significantly decreased in the prefrontal cortex (PFC) of postmortem tissues from ASD patients. Knockdown of *Ash1L* in PFC of juvenile mice induces the downregulation of risk genes associated with ASD, intellectual disability (ID) and epilepsy. These downregulated genes are enriched in excitatory and inhibitory synaptic function and have decreased H3K4me3 occupancy at their promoters. Furthermore, *Ash1L* deficiency in PFC causes the diminished GABAergic inhibition, enhanced glutamatergic transmission, and elevated PFC pyramidal neuronal excitability, which is associated with severe seizures and early mortality. Chemogenetic inhibition of PFC pyramidal neuronal activity, combined with the administration of GABA enhancer diazepam, rescues PFC synaptic imbalance and seizures, but not autistic social deficits or anxiety-like behaviors. These results have revealed the critical role of ASH1L in regulating synaptic gene expression and seizures, which provides insights into treatment strategies for *ASH1L*-associated brain diseases.

## Abstract (web summary)

*ASH1L* haploinsufficiency is strongly linked to autism, despite the unknown mechanism. Here, the authors show that *ASH1L* deficiency in prefrontal cortex causes the downregulation of synaptic genes, leading to seizures, which is rescued by chemogenetic and pharmacological restoration of excitation/inhibition balance.

## Introduction

Autism spectrum disorder (ASD) is a prevalent neurodevelopmental disorder with significant genetic heterogeneity and comorbidities, such as epilepsy and intellectual disability (ID)^1–3^. Large-scale human genetic studies have identified *ASH1L* as a convergent high-risk gene for ASD, epilepsy, and Tourette syndrome^2,4–6^. Over 100 loss-of-function (LOF) mutations in the coding regions of *ASH1L* have been discovered, and individuals harboring *ASH1L* mutations display a variety of symptoms associated with these brain disorders, including social deficits, repetitive behaviors, and seizures^3,6–9^. Despite the progress in genomics, it remains largely unknown on how *ASH1L* insufficiency contributes to the disease phenotypes.

ASH1L, the human homolog of Drosophila Ash1 (absent, small, or homeotic-like 1), is a histone methyltransferase catalyzing H3K4 and H3K36 methylation, which plays an important role in chromatin modification and gene transcription^10,11^. Whole-body homozygous deletion of *Ash1L* leads to lethality in mice^12,13^. ASH1L is enriched in excitatory and inhibitory neuronal lineages^2^, highly expressed in the prefrontal cortex (PFC) during prenatal development, persistently peaking at postnatal and in adulthood^14^. PFC is one of the key brain regions critical for cognitive, emotional, and social function^15–17^, and PFC dysfunction are strongly implicated in ASD patients^18^. Developmental trajectory of human PFC shows that the expression of synaptic genes in PFC peaks at juvenile to early adolescence (3.5–10 years old)^19–21^, a critical time window of phenotypic manifestation, diagnosis, and therapy for children with ASD, ID, and epilepsy. In this study, we examined the molecular, synaptic, and behavioral alterations resulting from *Ash1L* deficiency in PFC of mice during the juvenile to the early adolescent period (5–6 weeks old), matching the critical window of synaptic and behavioral development in humans. Our results have revealed the causal relationship between the Ash1L knockdown and altered synaptic gene expression, which leads to excitation/inhibition (E/I) imbalance and seizures. In addition, we have uncovered an intervention strategy to rescue seizures and early mortality induced by *Ash1L* deficiency in PFC.

## Results

**Fig. 1:**
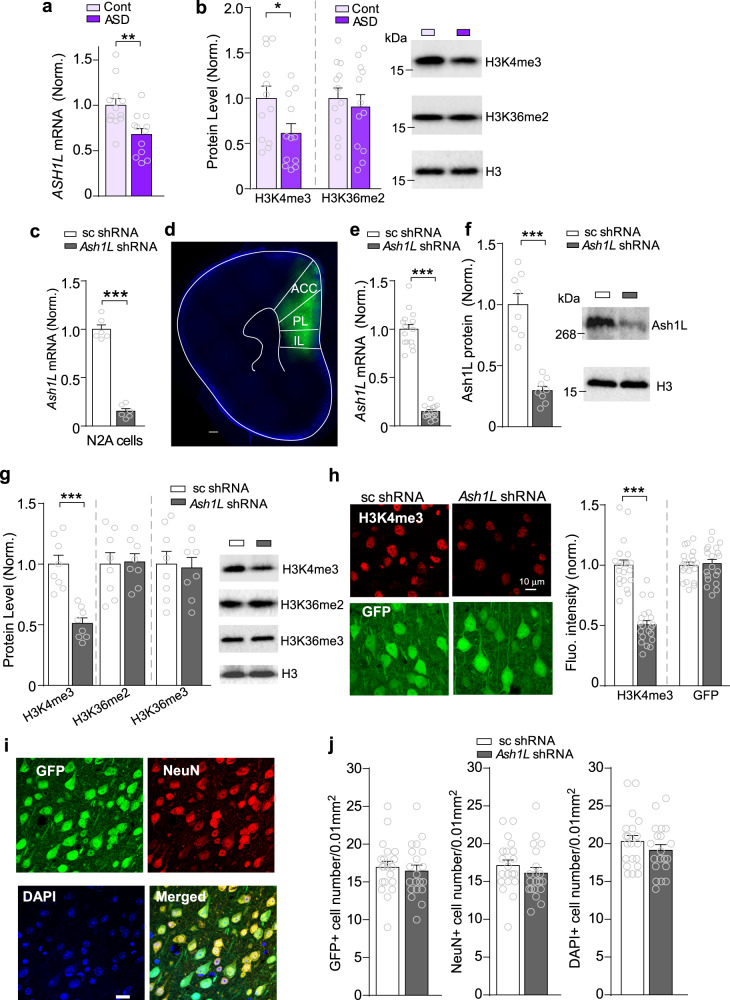
*ASH1L* expression and H3K4me3 level are significantly decreased in PFC of postmortem ASD patients, which is replicated by knockdown of *Ash1L* in PFC of young mice. **a** Quantitative PCR data showing *ASH1L* mRNA levels in postmortem PFC tissues from control humans vs. idiopathic ASD patients. *n* = 12 humans (10 M,2 F)/group, **p < 0.01, two-tailed *t* test. **b** Western blot data showing H3K4me3 and H3K36me2 levels in postmortem PFC from control vs. ASD patients. *n* = 12 humans(10 M,2 F)/group, *p < 0.05, two-tailed *t* test. **c** Quantitative PCR data showing *Ash1L* mRNA level in N2A cells transfected with *Ash1L* shRNA or scrambled (sc) shRNA. *n* = 6/group. ***p < 0.001, two-tailed *t* test. **d** A confocal image showing the viral-infected PFC region (stained with DAPI, blue) from a mouse with the stereotaxic injection of *Ash1L* shRNA AAV (GFP-tagged). Scale bar: 300 μm. **e**, **f** Quantitative PCR and Western blot data showing *Ash1L* mRNA and protein levels in PFC of mice (5-week-old) with the stereotaxic injection of *Ash1L* shRNA vs. sc shRNA AAV. *Ash1L* mRNA, *n* = 15 mice(8 M,7 F) for sc shRNA group, *n* = 14 mice(7 M,7 F) for *Ash1L* shRNA group; Ash1L protein, *n* = 8 mice(4 M,4 F)/group, ***p < 0.001, two-tailed *t* test. **g** Western blot data showing H3K4me3, H3K36me2, and H3K36me3 levels in PFC infected with *Ash1L* shRNA or scrambled shRNA AAV. *n* = 8 mice (4 M,4 F)/group, ***p < 0.001, two-tailed *t* test. **h** Representative confocal images and quantification of immunostaining of H3K4me3 (red) in PFC neurons infected with *Ash1L* shRNA or a scrambled shRNA AAV (GFP+ , green). *n* = 20 images/4 mice (2 M,2 F)/group, ***p < 0.001, two-tailed *t* test. Scale bar: 10 μm. **i**, **j** Re*p*resentative confocal images and quantification of immunostaining of NeuN (red) and DAPI (blue) in PFC neurons infected with *Ash1L* shRNA or a scrambled shRNA AAV (GFP+ , green). Slices were collected at 8–9 days postinfection. *n* = 20 images/4 mice (2 M,2 F)/group. Scale bar: 20 μm. All the full Western blots are included in Supplementary Fig. 7a–c. Data are presented as mean values ± SEM. Detailed statistical data are provided in a Source Data file.

ASH1L is recognized as a top-ranking risk factor for multiple brain diseases, including ASD^2^, Epilepsy^4,5^, and ID^22^. To find out whether ASH1L expression is altered in the disease condition, we examined the mRNA level of *ASH1L* in postmortem PFC tissues (Brodmann’s Area 9) from idiopathic autistic patients. As shown in Fig. 1a, *ASH1L* mRNA was significantly lower in PFC of autistic humans, compared to age- and sex-matched control subjects. Since ASH1L is a histone lysine methyltransferase with the specificity of H3K4 and H3K36 methylation^10,23–25^, we further examined these histone marks in human tissues. As shown in Fig. 1b, the level of H3K4me3 was significantly decreased in the PFC of autistic patients, while no significant change was found on the level of H3K36me2.

To investigate the biological function of ASH1L and its mechanistic link to brain disorders, we generated an *Ash1L* short hairpin RNA (shRNA) viral vector (GFP-tagged). The knockdown efficiency was confirmed by the significantly decreased level of *Ash1L* mRNA in N2A cells transfected with *Ash1L* shRNA (Fig. 1c). The *Ash1L* shRNA AAV was delivered to medial PFC bilaterally of young (5 weeks old) wild-type mice (Fig. 1d). In vivo knockdown was confirmed by the significantly lower level of *Ash1L* mRNA and Ash1l protein (333 KDa) in PFC of *Ash1L* shRNA AAV-injected mice (Fig. 1e, f). Western blots of the nuclear fraction of PFC indicated that the level of H3K4me3, but not H3K36me2 or H3K36me3, was significantly reduced by *Ash1L* knockdown (Fig. 1g). Immunostaining further demonstrated the significantly lower fluorescence intensity of H3K4me3 in GFP + cells infected with *Ash1L* shRNA AAV, compared to scrambled shRNA AAV (Fig. 1h). These data suggest that *Ash1L* deficiency diminishes the histone mark H3K4me3 that is linked to gene activation.

To find out the potential impact of *Ash1L* knockdown on the survival of PFC neurons, we performed immunohistochemical experiments with the neuronal marker NeuN. As shown in Fig. 1i, j, no significant loss of neurons was found in PFC from mice injected with *Ash1L* shRNA AAV.

### *Ash1l* deficiency in PFC induces the downregulation of genes associated with disease risk factors and enriched in synaptic function

**Fig. 2:**
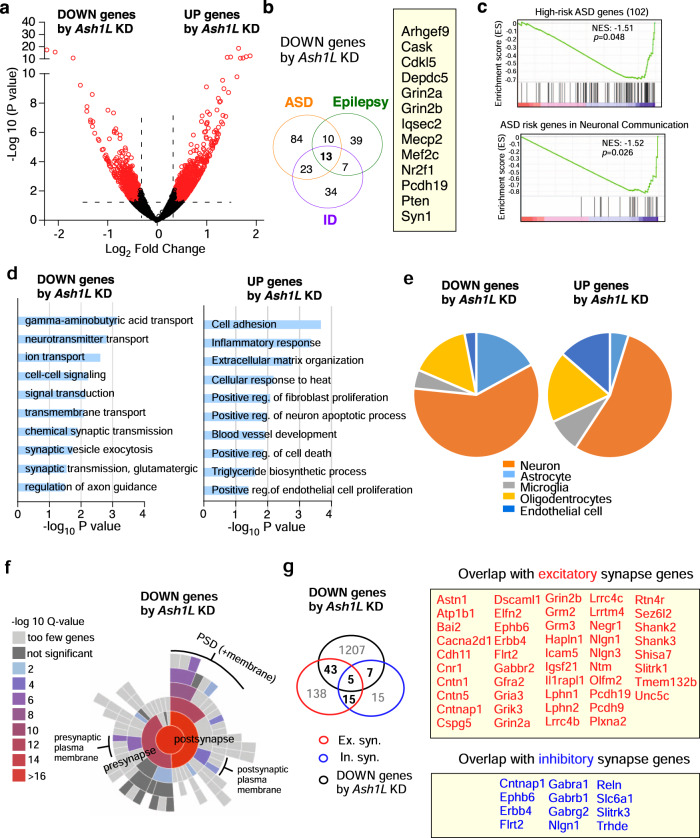
Genes downregulated by *Ash1L* deficiency in PFC overlap with risk factors for ASD, epilepsy, and ID, and are enriched in the regulation of synaptic homeostasis. **a** Volcano plot showing differentially expressed genes (DEGs) in PFC infected with *Ash1L* shRNA AAV, compared to scrambled shRNA AAV. **b** Venn diagram showing the overlapping of downregulated (DOWN) genes by *Ash1L* deficiency with the risk genes for ASD, epilepsy, and ID. Inset: the list of DOWN genes commonly occurring in all the three disorders. **c** Gene set enrichment analysis (GSEA) of the correlation between the DOWN genes by *Ash1L* deficiency and the 102 top-ranking ASD risk genes (top) or the top ASD risk genes involved in neuronal communication (bottom). **d** Gene Ontology analysis of downregulated or upregulated genes by *Ash1L* deficiency. **e** Pie chart showing the cell type distribution of *Ash1L* deficiency-induced DEG expression. **f** Sunburst plot representing cellular component enrichment analysis of downregulated synaptic genes by *Ash1L* deficiency. Higher red intensities are associated with more significant enrichments. All the identified synaptic genes are represented in the red circle at the center of the plot. **g** Venn diagram showing the overlapping of DOWN genes by *Ash1L* deficiency and genes at excitatory or inhibitory synapses. Inset: the list of downregulated synaptic genes.

Given the key role of histone methylation in gene regulation, we performed RNA sequencing (RNA-seq) to examine genome-wide gene expression changes induced by *Ash1L* deficiency in PFC. Numerous differentially expressed genes (DEGs) were identified in mice with *Ash1L* knockdown, compared to controls (Fig. 2a, 1262 genes downregulated, Supplementary Data 1; 1182 genes upregulated, Supplementary Data 2). To find out the link of these DEGs to ASH1L-associated brain disorders, we compared them with the gene databases for ASD (SFARI), epilepsy^4^ and ID^22^. As shown in Fig. 2b and Supplementary Data 3, a significant number of downregulated (DOWN) genes by *Ash1L* deficiency were disease risk factors (Supplementary Fig. 1a, SFARI ASD: 130, *P* = 5.67e–09; Supplementary Fig. 1c, epilepsy: 69, *P* = 2.69e–06; Supplementary Fig. 1d, ID: 77, *P* = 1.78e–04, hypergeometric test). The 13 DOWN genes by *Ash1L* deficiency commonly occurring in all the three disorders include *Grin2a/b* (NMDAR subunits), *Syn1* (presynaptic vesicle protein synapsin 1), *Mecp2* (reader of DNA methylation involved in gene silencing), and *Pten* (phosphatase dephosphorylating PIP_3_ and regulating Akt/PKB signaling pathway). The DOWN genes by *Ash1L* deficiency also overlapped significantly with the 102 ASD high-risk genes identified by recent large-scale human genetic studies (Supplementary Fig. 1b, 21, *P* = 3.6611e-05, hypergeometric test), which are enriched in gene expression regulation (GER), neuronal communication and cytoskeleton^2^. In contrast, upregulated (UP) genes by *Ash1L* deficiency were not enriched in disease risk factors (Supplementary Fig. 2, SFARI ASD: 45; epilepsy: 31; ID: 36, all under enriched). Gene Set Enrichment Analysis (GSEA) indicated that the ASD high-risk genes, particularly those involved in neuronal communication, were significantly correlated with the DOWN genes by *Ash1L* deficiency (Fig. 2c). These data suggest that Ash1L plays a central role in maintaining the transcription of multiple disease risk genes involved in synaptic function and neuronal signaling.

Gene Ontology analyses of DEGs by *Ash1L* deficiency (Fig. 2d) revealed that the DOWN genes were mostly enriched in biological processes involving gamma-aminobutyric acid (GABA) transport, ion transport, and glutamatergic synaptic transmission (Supplementary Data 4), while the UP genes were largely involved in cell adhesion, inflammatory response, and apoptosis (Supplementary Data 5). Mapping DEGs to markers of specific cell types^26^, we found that DOWN or UP genes by *Ash1L* deficiency were mainly in neurons, and some were in oligodendrocytes, astrocytes, or endothelial cells (Fig. 2e).

Given the enrichment of GO pathways involved in synaptic function for DOWN genes by *Ash1L* deficiency, we further compared them with SynGO, a synaptic gene ontology database^27^. Two hundred and thirteen of these DOWN DEGs were identified as synaptic genes. Synaptic enrichment analyses identified 16 cellular components and 26 biological processes among DOWN genes by *Ash1L* deficiency, with postsynaptic and presynaptic plasma membrane as the most enriched and abundant subcellular components (Fig. 2f). On the other hand, no enriched synaptic components or processes were present with UP genes by *Ash1L* deficiency (Supplementary Fig. 3a).

We further compared DOWN DEGs by *Ash1L* deficiency with synaptic cleft proteome^28^. We found 48 genes overlapping with excitatory synaptic proteins (including *Grm2/3, Grin2a/b, Gria3*, and *Shank2/3)*, 12 genes overlapping with inhibitory synaptic proteins (including *Gabra1/b1/g2* and *Slc6a1*), and 5 genes overlapping with dual-localized synaptic proteins (including *CntnAp1* and *Nlgn1*) (Fig. 2g, 55, *P* = 7.06e-15, hypergeometric test). In contrast, no significant overlapping was found among UP genes with excitatory or inhibitory synaptic proteins (Supplementary Fig. 3b).

**Fig. 3:**
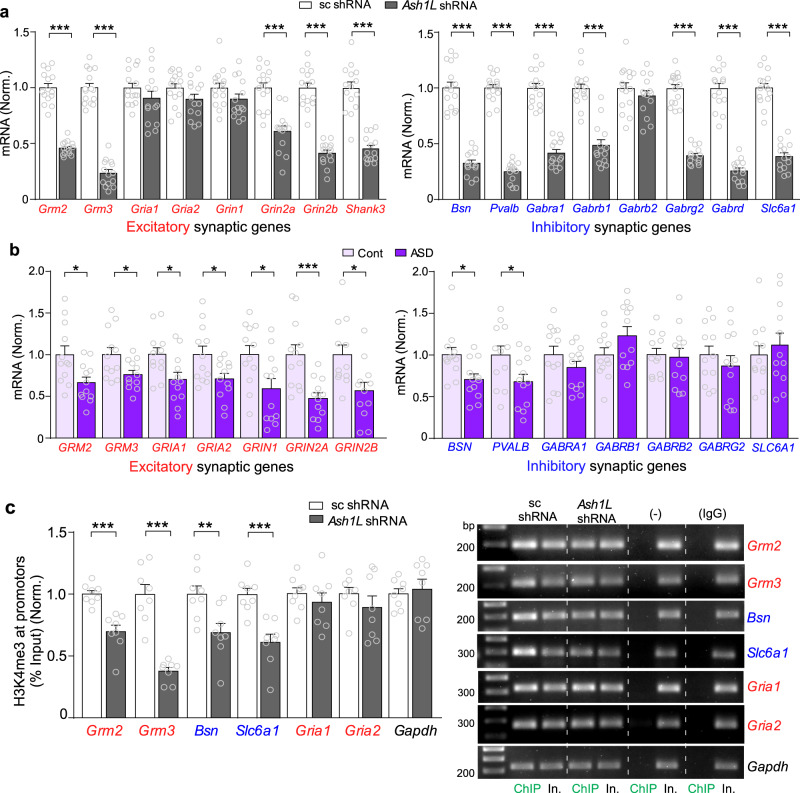
The transcriptional loss of synaptic genes by *Ash1L* deficiency are correlated with the reduced H3K4me3 enrichment at their promoters. **a**, **b** Quantitative real-time PCR showing the mRNA level of excitatory and inhibitory synaptic genes in PFC of mice infected with *Ash1L* shRNA vs. scrambled shRNA (**a**) or postmortem PFC tissues from ASD patients vs. control subjects (**b**). A, *n* = 14–15 mice (7–8 M,7 F)/group; B, *n* = 12 humans(10 M,2 F)/group, *p < 0.05, two-tailed *t* test. **c** ChIP assay showing the enrichment of H3K4me3 at the promoter region of *Grm2*, *Grm3*, *Bsn, Slc6a1*, and *Gapdh* in PFC of mice infected with *Ash1L* shRNA vs. scrambled shRNA AAV. *n* = 8 mice(4 M,4 F)/group. Inset: PCR images of the ChIP data. Rabbit IgG and omission of H3K4me3 antibody were used as the negative control. In.: input. All the full PCR gels are included in Supplementary Fig. 7d. Data are presented as mean values ± SEM. Detailed statistical data are provided in a Source Data file.

To confirm the RNAseq data, we performed qPCR to examine the expression of selected synaptic genes. As shown in Fig. 3a, the mRNA level of excitatory synaptic genes *Grm2/3* (encoding mGluR2/3), *Grin2a*/b (encoding NR2A/B), and *Shank3* (encoding the PSD scaffold protein Shank3) were significantly decreased in mice infected with *Ash1L* shRNA AAV, while *Grin1* (encoding NR1) and *Gria1/2* (encoding GluR1/2) were largely unchanged. The inhibitory synaptic genes *Bsn* (encoding presynaptic scaffold protein Bassoon), *Pvalb* (encoding Parvalbumin), *Gabra1/b1/g2* (encoding GABA_A_ receptor α1, β1 and γ2 subunits) and *Scl6a1* (encoding GABA transporter GAT-1) were also significantly decreased in *Ash1L*-deficient mice.

To find out the translational value of our mouse studies, we also examined the expression of synaptic genes in PFC (BA9) of human postmortem samples. A significant reduction of mRNA was found in autistic patients on excitatory synaptic genes *GRM2/3, GRIA1/2, GRIN1/2* *A/2B*, and inhibitory synaptic genes *BSN* and *PVALB*, but not *GABRA1/B1/B2/G2* (Fig. 3b). These data suggest that *Ash1L* deficiency in PFC induces the loss of selective synaptic genes at excitatory and inhibitory synapses, some of which are replicated in idiopathic autistic patients.

To find out whether the transcriptional inhibition of synaptic genes by *Ash1L* deficiency is linked to the reduction of permissive H3K4me3 level, which is required to maintain open chromatin states for activation of transcription^29,30^, we performed ChIP assays to examine H3K4me3 occupancy at the promoter region (±2Kb surrounding Transcription Start Site (TSS)) of these synaptic genes. As illustrated in the Genome browser snapshots of H3K4me1, H3K4me2, and H3K4me3 (GSE123652) at synaptic gene promoters (Supplementary Fig. 4), H3K4me3 has the most prominent occupancy. We found that H3K4me3 occupancy at promoters of *Grm2*, *Grm3, Bsn,* and *Slc6a1* was significantly reduced in *Ash1L* shRNA AAV-infected PFC, while no significant change was found on H3K4me3 occupancy at *Gria1, Gria2,* and *Gapdh* promoters (Fig. 3c). These data have linked the *Ash1L* deficiency-induced transcriptional loss of synaptic genes to H3K4me3 reduction in PFC.

### *Ash1L* deficiency in PFC causes neuronal hyperactivity and disrupts the balance of excitatory and inhibitory synaptic transmission

**Fig. 4:**
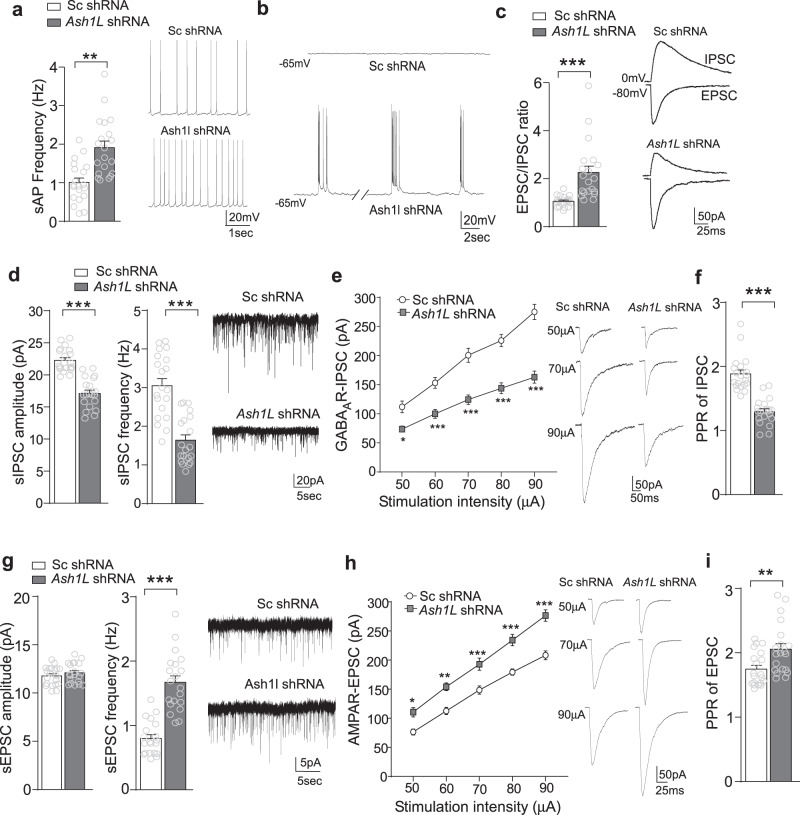
*Ash1L* deficiency induces the hyperactivity of PFC pyramidal neurons and disrupts the balance of excitatory and inhibitory synaptic responses. **a** Bar graphs showing the frequency of synaptic-driven spontaneous action potentials (sAP) in PFC pyramidal neurons from mice infected with scrambled (sc) shRNA vs. *Ash1L* shRNA AAV. *n* = 20 cells/4 mice (2 M,2 F)/group, *t*_38_ = 4.42, **p < 0.01, two-tailed t-test. Inset: representative sAP traces. **b** Representative traces showing the membrane excitability at the hyperpolarized level (−65 mV) in PFC pyramidal neurons infected with sc shRNA vs. *Ash1L* shRNA. **c** Bar graphs of evoked EPSC (recorded at −80 mV) to IPSC (recorded at 0 mV) ratio in PFC pyramidal neurons from mice infected with sc shRNA vs. *Ash1L* shRNA. *n* = 20 cells/4 mice (2 M,2 F)/group, *t*_38_ = 4.34, ***p < 0.001, two-tailed *t* test. Inset: representative EPSC and IPSC traces. **d**–**f** Bar graphs of spontaneous IPSC amplitude and frequency (**d**), input-output curves of evoked GABA_A_R-IPSC (**e**) and paired-pulse ratio (PPR) of IPSC (**f**) in PFC pyramidal neurons from mice infected with sc shRNA vs. *Ash1L* shRNA. *n* = 20 cells/4 mice (2 M,2 F)/group. **d**, *t*_38_ = 7.80 (amp), t_38_ = 6.19 (freq), ***p < 0.001, two-tailed *t* test; **e**, F_1,38(genotype)_ = 45.87, *p < 0.05, ***p < 0.001, two-way rmANOVA; **f**, *t*_38_ = 7.74, ***p < 0.001, two-tailed *t* test. Inset: representative sIPSC and eIPSC traces. **g**–**i** Bar graphs of spontaneous EPSC amplitude and frequency (**g**), input-output curves of evoked AMPAR-EPSC (**h**) and PPR of EPSC (**i**) in PFC pyramidal neurons from mice infected with sc shRNA vs. *Ash1L* shRNA. *n* = 20 cells/4 mice (2 M,2 F)/group, **g**, *t*_38_ = 7.52 (freq), ***p < 0.001, two-tailed *t* test; **h**, *F*_1,38(genotype)_ = 33.92, *p < 0.05, **p < 0.01, ***p < 0.001, two-way rmANOVA; **i**, *t*_38_ = 2.91, **p < 0.01, two-tailed t-test. Data are *p*resented as mean values ± SEM. Detailed statistical data are provided in a Source Data file.

Given the synaptic gene alterations by *Ash1L* deficiency, we next performed whole-cell patch-clamp recordings in PFC slices. Deep layer glutamatergic pyramidal neurons in PFC, which show the clearest deficits in autistic children^18^, were selected for electrophysiological measurements. As shown in Fig. 4a, PFC pyramidal neurons with A*sh1L* deficiency exhibited the significantly increased frequency of synaptic-driven, spontaneous AP (sAP). Even at the hyperpolarized potential (−65 mV) when control mice had no neurons with spikes (0/20 cells/4 mice), most A*sh1L*-deficient PFC pyramidal neurons exhibited sAP firing (20/20 cells/4 mice), and some even had rhythmic bursting (6/20 cells/4 mice, Fig. 4b). These data indicate that *Ash1L* deficiency causes the hyperactivity of PFC glutamatergic neurons, which could be due to the altered synaptic inputs and/or intrinsic excitability.

To find out the physiological basis of the hyperactivity by *Ash1L* deficiency, we examined excitatory and inhibitory synaptic responses. As shown in Fig. 4c, the ratio of excitatory postsynaptic currents (EPSCs) to inhibitory postsynaptic currents (IPSCs) was significantly increased in PFC pyramidal neurons infected with *Ash1L* shRNA AAV, suggesting that the E/I balance is switched to more excitation by *Ash1L* deficiency.

Further recordings of GABA_A_R-mediated IPSC indicated that *Ash1L* deficiency induced a significant decrease of the amplitude and frequency of spontaneous IPSC (Fig. 4d), the input/output curve of IPSC evoked by a series of stimulation intensities (Fig. 4e), as well as the paired-pulse ratio (PPR) of evoked IPSC (Fig. 4f), a readout of presynaptic transmitter release^31^. In parallel, recordings of AMPAR-mediated EPSC indicated that *Ash1L* deficiency induced a significant increase of the frequency, but not the amplitude, of spontaneous EPSC (Fig. 4g), the input/output curve of evoked EPSC (Fig. 4h), as well as PPR of EPSC (Fig. 4i). These results suggest that *Ash1L* deficiency leads to the depression of GABAergic inhibition and the enhancement of glutamatergic excitation in PFC pyramidal neurons, probably via a pre- and postsynaptic mechanism.

We further examined the impact of *Ash1L* deficiency on intrinsic excitability and properties of PFC pyramidal neurons. As shown in Supplementary Fig. 5a, the frequency of action potentials evoked by injected currents was significantly higher in PFC pyramidal neurons from mice injected with A*sh1L* shRNA AAV. Moreover, ASH1L knockdown induced a significant increase of input resistance and a significant decrease of action potential threshold without changing capacitance or resting membrane potential (Supplementary Fig. 5b–e). RNAseq analysis revealed that a number of genes encoding voltage-gated potassium channels and other ion channels, transporters and transporting ATPases were significantly downregulated by *Ash1L* deficiency (Supplementary Fig. 5f), which may underlie the elevated intrinsic excitability.

### *Ash1L* deficiency in PFC induces seizures, which is ameliorated by combined chemogenetic and pharmacological treatment to restore synaptic balance

**Fig. 5:**
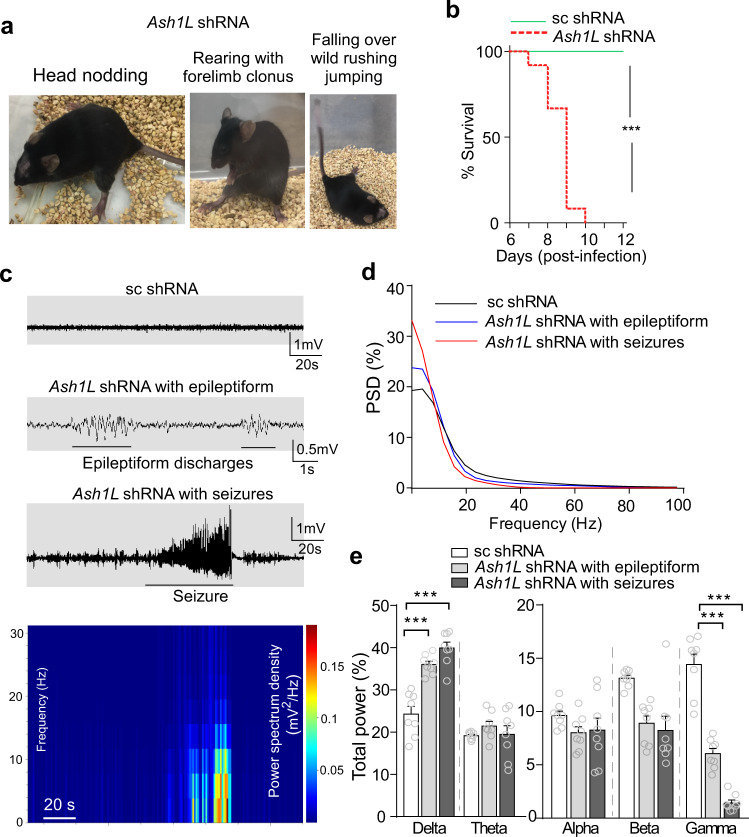
*Ash1L* deficiency in PFC induces epileptic seizures and mortality. **a** Snapshot pictures showing *Ash1L* shRNA-infected mice with seizures at different stages based on the Racine seizure behavior scoring paradigm. **b** Survival curves showing the mortality rates in mice infected with scrambled shRNA vs. *Ash1L* shRNA AAV. *n* = 20 mice(10 M,10 F)/group, ***p < 0.001, Mantel–Cox test. **c** Representative EEG traces from mice with PFC injection of scrambled shRNA or *Ash1L* shRNA AAV showing epileptiform discharges or seizures (high-frequency, high-voltage, rhythmic activity with clear onset, progression, and termination). Bottom: Spectrogram showing the frequency and power density (color scale, blue: 0; red: 0.2 mV2/Hz) before, during, and after seizures. **d**, **e** Comparison of total power spectral density (PSD, **d**) and the relative power in each EEG frequency band (**e**) between mice infected with scrambled shRNA vs. *Ash1L* shRNA AAV. EEG band: Delta (0.1–4 Hz), theta (4–8 Hz), Alpha (8–13 Hz), beta (13–30 Hz), and gamma (30–60 Hz). *n* = 8 mice(4 M,4 F)/group, ***p < 0.001; **p < 0.01, two-tailed *t* test. Data are presented as mean values ± SEM. Detailed statistical data are provided in a Source Data file.

Next, we examined behavioral phenotypes by *Ash1L* deficiency in PFC. We found that mice with PFC injection of *Ash1L* shRNA AAV displayed epileptic phenotypes, as indicated by the different stages of seizures (Fig. 5a) based on the Racine seizure behavior scoring paradigm^32^. These mice usually died at 7-9 days after viral injection (Fig. 5b). Dead mice displayed a typical pose with clenched forelimbs and stretched-out hind limbs, indicating that they died from status epilepticus. EEG recordings from PFC of mice with *Ash1L* deficiency showed epileptiform activity (an interictal marker of epilepsy) at 1–2 days before overt seizures, and high amplitude spike- and wave-complexes while displaying seizures (Fig. 5c). The percentage total power of delta frequency was significantly increased, and the gamma frequency was significantly decreased in *Ash1L* deficiency mice with epileptiform activity and with seizures (Fig. 5d, e).

The seizure phenotype could be due to E/I imbalance resulting from depressed GABAergic inhibition and enhanced glutamatergic excitation by *Ash1L* deficiency. One treatment strategy is to use the GABAAR positive allosteric modulator diazepam (DZ) to enhance GABAergic inhibition. Another treatment strategy is to use CaMKII-driven Gi-coupled DREADD (Designer Receptors Exclusively Activated by Designer Drugs) to inhibit pyramidal neuron activity. Gi-DREADD is activated by exogenous administration of the small molecule clozapine-N-oxide (CNO), which can induce neuronal silencing^33^. This chemogenetic technology has been used to suppress seizures^34,35^.

**Fig. 6:**
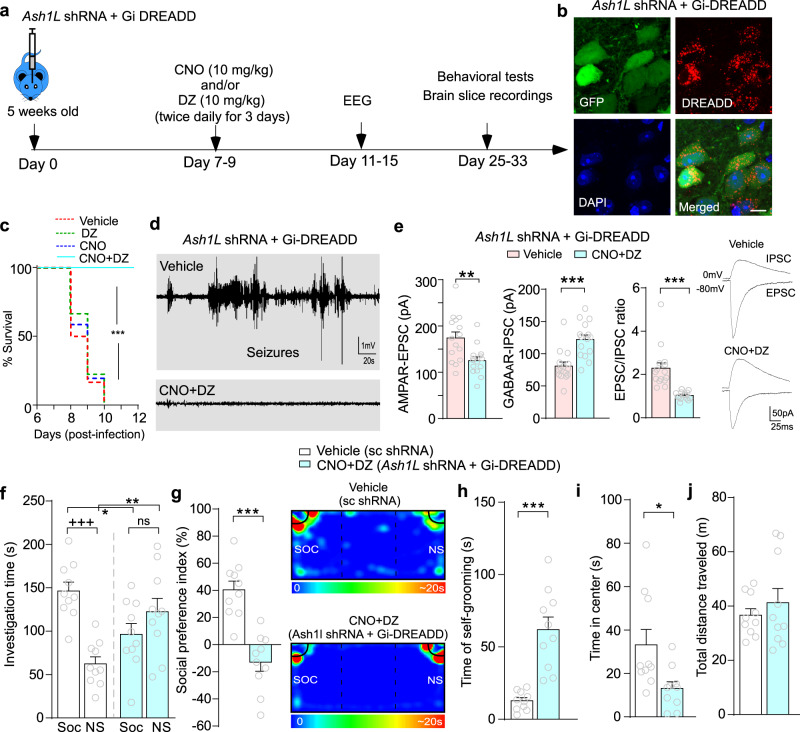
Combined chemogenetic and pharmacological treatment of *Ash1L*-deficient mice ameliorates seizures, but not other ASD-related behavioral deficits. **a** A schematic diagram of the timeline for treatment and experimental measurements. **b** Confocal images showing CaMKII-driven Gi-DREADD (red) expression in *Ash1L* shRNA (Green)-infected PFC pyramidal neurons. Scale bar: 10 μm. **c** Survival curves showing the mortality rates of *Ash1L-*deficient mice (w/Gi-DREADD) treated with vehicle or CNO + diazepam (DZ). **d** Representative EEG traces from *Ash1L-*deficient mice treated with vehicle, DZ alone, CNO alone, or CNO + DZ. *n* = 12 mice(6 M,6 F)/group, ***p < 0.001, Mantel-Cox test. **e** Bar graphs showing EPSC, IPSC, and EPSC/IPSC ratio recorded in PFC pyramidal neurons from *Ash1L-*deficient mice treated with vehicle or CNO + DZ. *n* = 15 cells/4 mice (2 M,2 F)/group, **p < 0.01, ***p < 0.001, two-tailed *t* test. **f**, **g** Bar graphs showing the amount of time spent on interacting with the social (Soc) vs. nonsocial (NS) stimulus (**f**) and social preference index (**g**) in 3-chamber social preference tests of *Ash1L-*deficient mice (w/Gi-DREADD) treated with CNO + DZ vs. control mice (infected with scrambled shRNA) treated with vehicle. *n* = 10 mice(5 M,5 F)/group, f, *p < 0.05, **p < 0.01, +++*p* < 0.001 (Soc vs. NS), two-way ANOVA; **g**, *t*_18_ = 5.75, ***p < 0.001, two-tailed *t* test. Inset: representative heatmaps illustrating the time spent in different locations of the 3 chambers (color scale, blue: 0 sec; red: ~20 sec). **h**–**j** Bar graphs showing the time spent in self-grooming (**h**), the time in center of open field tests (i), and the distance traveled in locomotion tests (**j**) of *Ash1L-*deficient mice treated with CNO + DZ vs. control mice treated with vehicle. *n* = 10 mice (5 M,5 F)/group, **h**, *t*_18_ = 5.48, ***p < 0.001; **i**, *t*_18_ = 2.6, *p < 0.05, two-tailed *t* test. Data are presented as mean values ± SEM. Detailed statistical data are provided in a Source Data file.

To test this, we co-injected Gi-DREADD and *Ash1L* shRNA AAV to the PFC of mice. A timeline of treatment is illustrated in Fig. 6a. As shown in Fig. 6b, most of DREADD-expressing pyramid neurons had *Ash1L* knockdown (96 ± 1.5% co-infection, *n* = 3 mice). Mice infected with *Ash1L* shRNA and Gi-DREADD displayed seizures at 7–9 days post-injection. Administration of DZ (10 mg/kg, i.p. twice daily for 3 days) failed to ameliorate mortality in *Ash1L-*deficient mice (Fig. 6c), suggesting that targeting the GABA system alone is not sufficient. Administration of CNO (10 mg/kg, i.p. twice daily for 3 days) alone was also incapable of limiting mortality (Fig. 6c). However, after the dual treatment with CNO and DZ, the high mortality of *Ash1L*-deficient mice was completely reversed (Fig. 6c). EEG showed that the seizure-associated high amplitude spike- and wave-complexes were terminated by CNO + DZ treatment (Fig. 6d). Patch-clamp recordings showed that the dual treatment significantly reduced AMPAR-mediated EPSC, enhanced GABA_A_R-mediated IPSC, and decreased EPSC/IPSC ratio to the normal range (Fig. 6e). These results suggest that seizures and E/I imbalance induced by *Ash1L* deficiency could be mitigated by the combined excitatory neuron activity suppression and inhibitory synaptic transmission potentiation.

The elimination of seizures by CNO + DZ treatment enabled us to test other behavioral phenotypes in mice with *Ash1L* deficiency in PFC. The two core symptoms of ASD, social deficits and repetitive behaviors, were first examined. Compared to the vehicle control group, in the three-chamber social preference test^36^, mice with *Ash1L* deficiency spent significant less time interacting with the social stimulus and significant more time interacting with the nonsocial stimulus, consequently had a significant loss of social preference index (Fig. 6f, g). Moreover, *Ash1L*-deficient mice spent significantly more time on self-grooming (Fig. 6h), a rodent behavior thought to model repetitive behaviors observed in human ASD patients^37^. In the open-field test, *Ash1L*-deficient mice spent significant less time in the center, suggesting increased anxiety (Fig. 6i), while they did not exhibit significant changes on locomotion (Fig. 6j). CNO or DZ did not affect sociability, repetitive behavior, anxiety, or locomotion in the vehicle control group (Supplementary Fig. 6), suggesting that the phenotypes of *Ash1L*-deficient mice are unlikely due to the pharmacological treatment. These data indicate that mice with *Ash1L* deficiency in PFC exhibit ASD-like behavioral deficits beyond seizures, which could not be ameliorated by CNO + DZ treatment.

## Discussion

Histone methylation plays a key role in maintaining transcriptional homeostasis via the activation and repression of gene transcription^29,38^. Histone methyltransferases and demethylases that control histone methylation have been identified as the most prominent ASD risk genes^1–3,8,39^. One of the histone methyltransferases, *ASH1L*, is a top-ranking ASD risk factor, and LOF mutations of *ASH1L* causes the manifestation of phenotypes associated with neurodevelopmental disorders^1,3,7,40–44^. ASH1L contains a conserved SET domain and acts as an activator of target genes by conferring a relaxed chromatin structure via H3K4 and H3K36 methylation^10,13,45^. Ash1L epigenetically affects the expression of osteogenic and chondrogenic transcription factors via modifying the enrichment of H3K4me3 on their promoter regions^46^. However, the role of Ash1L in regulating neuronal genes is largely unknown. Here we have found the significantly decreased expression of *ASH1L* and H3K4me3 in postmortem PFC tissues from idiopathic ASD patients, which may contribute to the alteration of H3K4me3 landscapes in PFC of ASD patients^47^.

Our transcriptomic analyses have identified a significant effect of *Ash1L* deficiency in PFC on risk genes associated with ASD, epilepsy, and ID. Downregulated genes by loss of *Ash1L* are enriched in neuronal communication/synaptic function and chromatin remodeling/gene transcription, such as *Grin2b*, *Slc6a1*, *Shank2/3, Pten*, *Arid1b*, *Bcl11a*, and *Foxp1/2*, which are also high-risk autism genes identified by transcriptomic analysis of autistic human brains^2,48^. It suggests that *Ash1L* deficiency in PFC leads to disrupted synaptic homeostasis, a key pathophysiological mechanism of ASD and related neurodevelopmental disorders^2,49–54^. The decreased transcription of synaptic genes by *Ash1L* deficiency is correlated with the decreased H3K4me3 occupancy at their promoters from our ChIP data. Consistently, in human PFC during early postnatal development, neuron-specific H3K4me3 peaks are enriched in synaptic transmission components^55^. H3K4me3 has a much higher level of enrichment at synaptic gene promoters than H3K4me1 or H3K4me2.

Comparing our RNAseq data with the ASH1L genomics datasets^56,57^, we did not find much overlapped target genes, probably because both of the prior studies used neural progenitor cell lines, which do not have the well-developed expression of synaptic genes as the mature cortical neurons that we used from young adult mice. However, when comparing the GO pathways between the Ash1L KD LUHME model^57^ and our Ash1L KD mouse model, there is a general overlap involved in “synaptic transmission” and “synapse organization”.

Aberrant synaptic function is thought to be a major pathogenic factor in ASD^1,58^. Our physiological results have confirmed the synaptic dysfunction by *Ash1L* deficiency. The increased E/I ratio leads to hyperactivity of PFC pyramidal neurons. The diminished synaptic inhibition by *Ash1L* deficiency could result from the loss of GABA synapses or GABAergic interneurons or GABA_A_ receptors because of the reduced expression of inhibitory synaptic genes *Bsn*, *Pvalb*, and *Gabra1/b1/g2*. On the other hand, the elevated synaptic excitation by *Ash1L* deficiency could result from the loss of mGluR2/3-mediated inhibition of glutamate release because of the reduced expression of excitatory synaptic genes *Grm2* and *Grm3*. In addition to synaptic imbalance, *Ash1L* deficiency-induced hyperactivity of PFC glutamatergic neurons may also be attributable to the elevated intrinsic excitability, which could result from the downregulation of genes encoding voltage-gated potassium channels and transporters.

The elevated PFC pyramidal neuronal excitability, increased E/I ratio, and excessive synchronized cortical network activity of *Ash1L*-deficient mice is linked to seizures, which recapitulates the phenotype of some autistic children carrying *ASH1L* variants^8^. Among ASD children aged 2 to 17, up to 40% have epilepsy, and up to 80% present epileptiform or EEG abnormalities^59–61^. A latest clinical report also shows that a de novo *ASH1L* truncating mutation causes seizures (refractory epilepsies) in twin sisters^62^. The diminished GABAergic inhibition and elevated glutamatergic excitation by *Ash1L* deficiency prompted us to target these systems for therapeutic intervention. The GABA_A_R positive allosteric modulator Diazepam (DZ), which has been commonly used to treat seizures^63^, is insufficient to mitigate seizures in *Ash1L*-deficient mice, consistent with the reports that most of the epileptic patients with ASD remain refractory to available medications^34,64^. So we combined DZ with hM4Di DREADD to dampen glutamatergic neuronal activity, which can suppress spontaneous seizures in mouse models for epilepsy^34,64^. Remarkably, the dual treatment ameliorated seizures, PFC hyperactivity, and synaptic imbalance induced by *Ash1L* deficiency. Moreover, the effect of CNO + DZ is long-lasting, with no recurring seizures after the 3-day dual treatment over 4–6 months. The reason for this prolonged therapeutic effect is not known. We speculate that seizure sensitivity is higher in the adolescent stage (5–6 weeks old) when PFC is still under development. Once seizures are prevented from occurring at the young age with CNO + DZ treatment, the restored E/I balance in PFC keeps adult mice devoid of seizures. Whether these treated mice with *Ash1L* deficiency are still prone to seizure-inducing stimuli awaits to be further studied.

After elimination of seizures, mice with the PFC knockdown of *Ash1L* exhibit significant social preference deficits, repetitive grooming, and anxiety-related behaviors, all of which are consistent with the phenotypes of a mouse model with *Ash1L* knockout in the developing brain^56^. The pathophysiological mechanisms underlying these autism-like behaviors caused by *Ash1L* deficiency and therapeutic strategies to rescue these abnormalities will be investigated in future studies.

**Fig. 7:**
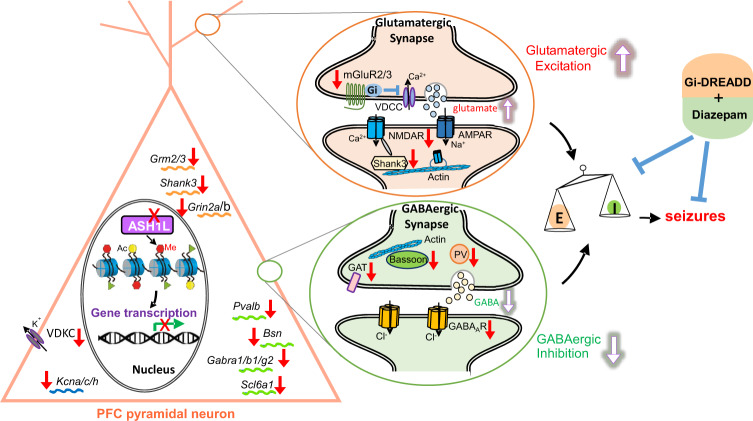
A schematic model showing the causal relationship between *Ash1L* deficiency and altered synaptic gene expression and neuronal excitability. Knockdown of *Ash1L*, a histone methyltransferase catalyzing the permissive H3K4me3, causes the reduced H3K4me3 at synaptic gene promoters, leading to the reduced transcription of excitatory synaptic genes (e.g., *Grm2/3, Grin2a/b, Shank3*) and inhibitory synaptic genes (e.g., *Bsn*, *Pvalb*, *Gabra1/b1/g2, Scl6a1*). Consequently, glutamatergic transmission is elevated, which is likely due to the loss of Gi-coupled mGluR2/3-mediated inhibition of voltage-dependent calcium channels (VDCC) and glutamate release^70^, while GABAergic transmission is diminished, which is likely attributable to the loss of GABAergic synapses or postsynaptic GABA_A_Rs. The E/I balance is switched to more excitation, which induces severe seizures and early mortality. Treatment with the Gi-DREADD and GABA_A_R positive allosteric modulator diazepam can ameliorate E/I imbalance and limit seizures.

In summary, our results have revealed a causal relationship between the deficiency of *Ash1L* and the dysregulation of synaptic gene transcription and the alteration of neuronal excitability (Fig. 7). It provides a mechanistic framework to understand how the three sets of genetic disruptions commonly found in autism (synaptic, transcriptional, and chromatin genes)^1^ are potentially interconnected.

## Methods

### Animals, human postmortem tissues, and compounds

All experiments were performed with the approval of the institutional animal care and use committee (IACUC) in State University of New York at Buffalo. Wild-type (WT) mice (5 weeks old, male and female) with C57BL/6 J background were used in this study. Mice were group-housed (temperature: 72 °F; humidity: 56%) with *ad libitum* food accessibility in the 12-hr light-dark cycle (light: 6 am–6 pm; dark: 6 pm–6 am) and Experiments were carried out by investigators in a blinded fashion (with no prior knowledge of treatments).

Frozen human postmortem tissues (Brodmann’s Area 9) from autism patients and age-matched healthy controls were provided by NIH NeuroBioBank. Detailed information about these ASD patients is included in Supplementary Data 6. Our cohort was composed of 12 humans with autism (10 males, 2 females) and 12 unaffected controls (10 males, 2 females). The median age at death was 12 years (range 5–19). Upon arrival, tissue was stored in a −80 °C freezer until used for RNA and protein extraction^65^. This study complied with all relevant ethical regulations for work with human participants and was approved by the NIH NeurobioBank Review Board.

Clozapine-*N*-oxide dihydrochloride (CNO) (Tocris) or diazepam (Sigma) was dissolved in saline or dimethyl sulfoxide (DMSO) to make the stock solution (10 mg/ml) and stored at −20 °C. Before use, the stock solution was diluted with saline. Each injection was 10 mg/kg of body weight.

### Virus generation and delivery

To knockdown *Ash1L*, short-hairpin RNA (shRNA) sequence (GCTGTGTGTTGGACCTTTATA) was cloned into GFP-tagged adeno-associated virus (AAV) vector (Addgene) under the control of U6 promotor. Viral particles (titer: 1.3 × 10^14^ vg/ml) were produced by the viral core center of Emory University. Ash1L shRNA AAV was bilaterally injected to the medial PFC (2.0 mm anterior to bregma; 0.25 mm lateral; 2.0 mm deep; 0.5 μl each side) of WT mice (5 weeks old) as we described before^66,67^. In brief, mice were anesthetized and placed on a stereotaxic apparatus (David Kopf Instruments, Tujunga, CA). The injection was carried out with a Hamilton syringe (needle gauge 31) at a speed of ~0.1 μl/min and the needle were kept in place for an additional 5 min. In some experiments, a CaMKII-driven hM4Di (Gi) DREADD and Ash1L shRNA (1:1 mixture) were injected into mPFC.

### Quantitative real-time RT-PCR

Total RNA was isolated from mouse PFC punches or human postmortem tissues using Trizol reagent (Invitrogen) and treated with DNase I (Invitrogen) to remove genomic DNA. Then iScriptTM cDNA synthesis Kit (Bio-Rad) was used to obtain cDNA from the tissue mRNA. Quantitative real-time PCR was carried out using the iCycler iQ™ RealTime PCR Detection System and iQ™ Supermix (Bio-Rad) according to manufacturer’s instructions. In brief, GAPDH was used as the housekeeping gene for quantitation of the expression of target genes in samples from mice infected with scrambled shRNA or Ash1L shRNA AAV. Fold changes in the target genes were determined by: Fold change = 2^−∆(∆CT)^, where ∆CT = C_T_(target) − C_T_(GAPDH), and ∆(∆C_T_) = ∆C_T_(Ash1L shRNA) − ∆C_T_(scrambled shRNA) or ∆(∆C_T_) = ∆C_T_(ASD patients) − ∆C_T_(Control). C_T_ (threshold cycle) is defined as the fractional cycle number at which the fluorescence reaches 10x of the standard deviation of the baseline. A total reaction mixture of 20 μl was amplified in a 96-well thin-wall PCR plate (Bio-Rad) using the following PCR cycling parameters: 95 °C for 5 min followed by 40 cycles of 95 °C for 45 s, 55 °C for 45 s, and 72 °C for 45 s. Primers for all target genes are listed in Supplementary Data 7.

### Western blotting of nuclear proteins

Nuclear extracts from mouse brains or human postmortem tissues were prepared according to the manufacturer’s instructions (Life Technologies) with modifications. Briefly, PFC punches from mouse slices containing GFP fluorescence or human postmortem tissues were collected, and then homogenized with 500 μl hypotonic buffer (20 mM Tris-HCl, pH 7.4, 10 mM NaCl, 3 mM MgCl_2_, 0.5% NP-40, 1 mM PMSF, with cocktail protease inhibitor). The homogenate was incubated on ice for 15 min and followed by centrifugation at 3,000 g, 4 °C for 10 min. The nuclear pellet was resuspended in 50 μl nuclear extract buffer (100 mM Tris-HCl, pH 7.4, 100 mM NaCl, 1 mM EDTA, 1% Triton X-100, 0.1% SDS, 10% glycerol, 1 mM PMSF, with cocktail protease inhibitor) and incubated on ice for 30 min with periodic vortexing to resuspend the pellet. After centrifugation, the supernatant for nuclear fractions was collected, boiled in 2 × SDS loading buffer for 5 min, and then separated on 6 or 12% SDS-polyacrylamide gels. Western blotting experiments for nuclear proteins were performed with antibodies against Ash1L (1:1000, LSBio, LS-B11718), H3K4me3 (1:1000, Cell Signaling, 9751), H3K36me2 (1:1000, Cell Signaling, 2901), H3K36me3 (1:1000, Cell Signaling, 9763), and Histone 3 (1:500, Cell Signaling, 4499).

### Chromatin immunoprecipitation (ChIP)

Briefly, PFC punches from mouse slices containing GFP fluorescence were collected. The dissected brain tissues from three mice were pooled as a single sample. Each sample was homogenized in 250 μl ice-cold douncing buffer (10 mM Tris-HCl, pH 7.5, 4 mM MgCl_2_,1 mM CaCl_2_). The homogenized sample was incubated with 12.5 μl micrococcal nuclease (5 U/ml, Sigma, N5386) for 7 min and terminated by adding EDTA at a final concentration of 10 mM. Then, hypotonic lysis buffer (1 ml) was added and incubated on ice for 1 hr. The supernatant was transferred to a new tube after centrifugation. After adding 10× incubation buffer (50 mM EDTA, 200 mM Tris-HCl, 500 mM NaCl), 10% of the supernatant was saved to serve as input control. To reduce nonspecific background, the supernatant was pre-cleared with 100 μl of salmon sperm DNA/protein A agarose-50% slurry (Millipore, 16–157) for 2 h at 4 °C with agitation. The pre-cleared supernatant was incubated with antibodies against H3K4me3 (8 μg per reaction; ab8580, Abcam) overnight at 4 °C under constant rotation, following by incubation with 60 μl of Salmon Sperm DNA/Protein A agarose 50% Slurry for 2 h at 4 °C. After washing for five times, bound complex was eluted twice from the beads by incubating with the elution buffer (100 μl) at room temperature. Proteins and RNA were removed by using proteinase K (Invitrogen) and RNase (Roche). Then, immunoprecipitated DNA and input DNA were purified by QIAquick PCR purification Kit (Qiagen). Quantification of ChIP signals was calculated as percent input. Purified DNA was subjected to qPCR reactions with primers against mouse *Grm2* promoter (Forward, 463 bp to 482 bp relative to TSS, 5′-GTCTTCAACCCTGATCCTCT-3′; Reverse, 632 bp to 651 bp relative to TSS, 5′-CTTGGGATTGGTAAGGAACT-3′), *Grm3* promoter (Forward, −482 bp to −461 bp relative to TSS, 5′-ATTCACCAACCTTTGTATGC-3′; Reverse, −253 bp to −232 bp relative to TSS, 5′-CCTCATTCTTCTCCTTTCCT-3′), *Slc6a1* promoter (Forward, 700 bp to 719 bp relative to TSS, 5′-CCAATGTCCTCTCAAAACAG -3′; Reverse, 869 bp to 888 bp relative to TSS, 5′-GCTAGCATCAAGTTCCACTC -3′), *Bsn* promoter (Forward, −462 bp to −444 bp relative to TSS, 5′-ATGTGGGCTCCTAACTCAC-3′; Reverse, −296 bp to −278bp relative to TSS, 5′-TACGTCTGTAATGCCAGTCA -3′), *Gria1* promoter (Forward, 738 bp to 757 bp relative to TSS, 5′-AACAGGGTTCAGAGTGCTTA -3′; Reverse, 931 bp to 950 bp relative to TSS, 5′-AATGCAATCAACCTAGCATC-3′), *Gria2* promoter (Forward, −496 bp to −477 bp relative to TSS, 5′-ATAGCAACCGGAAATCAGTT-3′; Reverse, −366 bp to −345 bp relative to TSS, 5′-GCCTCTTAGAGACCTCCAGT-3′), *Gapdh* promoter (Forward, −1192 bp to −1171 bp relative to TSS, 5′-TGTGCCCCATAACACAGCAT-3′; Reverse, −997 bp to −976 bp relative to TSS, 5′-TCCTGCAGACCCCTTAGTGT-3′).

### RNA-sequencing and bioinformatics analyses

Total RNA was isolated from mouse PFC punches containing GFP fluorescence using the RNAeasy Mini kit coupled to an RNase-free DNase step (Qiagen). The dissected brain tissues from three mice were pooled as a single sample. The RNA-seq libraries were constructed by TruSeq stranded total RNA plus Ribo-Zero kits (Illumina). Sequencing was carried out with the HiSeq 2500 platform (Illumina) at the Genomics and Bioinformatics Core of the State University of New York at Buffalo.

Raw fastq paired-end sequencing reads were aligned to the mouse reference genome mm10 using RNA STAR (Galaxy version 2.7.5b). Gene expression of mapped reads were then measured using featureCounts (Galaxy Version 1.6.4 + galaxy1), which produces compatible gene expression matrices. We then used DEseq2 (Galaxy Version 2.11.40.2) for differential gene expression analysis under default settings with featureCounts data as input. The differentially expressed genes (DEGs) between genotypes were defined with at least 1.2-fold change (FC) and P < 0.05 (compared to controls). GO analyses was performed using DAVID Functional Annotation Bioinformatics Microarray Analysis^68^.

### Immunohistochemistry

Mice were anesthetized and transcardially perfused with PBS, followed by 4% paraformaldehyde before brain removal. Brains were post-fixed in 4% PFA overnight and cut into 100 μm slices coronally. Slices were washed and blocked for 1 h in PBS containing 5% BSA and 0.05% Triton. After washing, slices were incubated with the primary antibody against H3K4me3 (1:500, Cell Signaling, 9751) or NeuN (1:1000, Millipore, MAB377) overnight at 4 °C. After washing three times in PBS, slices were incubated with secondary antibodies (Alexa Fluor 594, Invitrogen A11037) for 1 h at room temperature, followed by three washes with PBS. Slices were mounted on slides with Vectashield mounting media (Vector Laboratories). Images were acquired using a 63 × objective on a Leica TCS SP8 confocal microscope. All specimens were imaged under identical conditions and analyzed with identical parameters using Image J software (version 1.52d, NIH).

### Electrophysiological recordings of brain slices

Whole-cell voltage-clamp recording technique was used to measure synaptic currents in layer five pyramidal neurons of prefrontal cortical slices, as previously described^67,69^. Mouse slices (300 μm) were positioned in a perfusion chamber attached to the fixed stage of an upright microscope (Olympus) and submerged in continuously flowing oxygenated ACSF (in mM: 130 NaCl, 26 NaHCO_3_, 1 CaCl_2_, 5 MgCl_2_, 3 KCl, 1.25 NaH_2_PO_4_, 10 glucose, pH 7.4, 300 mOsm). Layer V mPFC pyramidal neurons were visualized with a 40X water-immersion lens and recorded with the Multiclamp 700 A amplifier (Molecular Devices, Sunnyvale, CA) and Clampex software 9 (Molecular Devices, Sunnyvale, CA).

For spontaneous inhibitory postsynaptic current (sIPSC) recording, neurons were held at −70 mV, and 25 μM CNQX was added to ACSF. Recording pipette contained the following internal solution (in mM: 100 CsCl, 30 N-methyl-D-glucamine, 10 HEPES, 4 NaCl, 1 MgCl_2_, 5 EGTA, 2 QX-314, 12 phosphocreatine, 5 MgATP, 0.5 Na_2_GTP, pH 7.2–7.3, 265–270 mOsm). For spontaneous excitatory postsynaptic current (sEPSC) recording, neurons were held at −70 mV, and 25 μM bicuculline was added to ACSF. Recording electrodes contained the following internal solution (in mM: 130 Cs-methanesulfonate, 10 CsCl, 4 NaCl, 10 HEPES, 1 MgCl_2_, 5 EGTA, 2 QX-314, 12 phosphocreatine, 5 MgATP, 0.2 Na_2_GTP, 0.1 leupeptin, pH 7.2–7.3, 265–270 mOsm).

Since sIPSC and sEPSC were measured in glutamate and GABA blockers respectively, which could miss network-level compensation of basal synaptic inhibition, E/I balance was measured by recording evoked AMPAR-EPSC (at −80 mV) and GABA_A_R-IPSC (at 0 mV) in the absence of blockers. Evoked synaptic current was generated with a pulse from a stimulation isolation unit controlled by a S48 pulse generator (Grass Technologies, West Warwick, RI). A bipolar stimulating electrode (FHC, Bowdoinham, ME) was placed ~100 μm from the neuron under recording. The same stimulation pulse (0.4 ms, 70 μA) was used for evoked EPSC and IPSC recordings. For input-output responses, synaptic current was elicited by a series of pulses with different stimulation intensities (50–90 μA) delivered at 0.05 Hz. For paired-pulse ratios, AMPAR-EPSC or GABA_A_R-IPSC was evoked by double pulses with a 50 ms interval.

To record the synaptic-driven spontaneous action potential (sAP), slices were bathed in a modified ACSF with low (0.5 mM) MgCl_2_ to elevate neuronal activity, which more closely mimics the ionic composition of the brain interstitial fluid in situ. No AMPA or GABA_A_ receptor blockers were added in sAP recordings. Whole-cell current-clamp techniques were used to measure action potential firing with the internal solution containing (in mM: 20 KCl, 100 K-gluconate, 10 HEPES, 4 ATP, 0.5 GTP, and 10 phosphocreatine). A small depolarizing current was applied to adjust the inter-spike potential to −60 to −65 mV. For the recording of evoked action potentials (eAP), AMPA and GABA_A_ receptor blockers were added to the ACSF, and a series of currents (−20 to 120 pA, 20 pA increment, 600 ms) was injected.

### EEG recording and power spectrum analyses

Mice were anesthetized with a mixture of ketamine (100 mg/kg, i.p.) and xylazine (5 mg/kg, i.p.) and positioned in a stereotaxic frame. After viral delivery to mPFC, a recording screw electrode was implanted in the prefrontal cortical surface (2.0 mm anterior and 0.25 mm lateral to bregma). A reference screw electrode and a ground electrode were placed in the left and right posterior cortex (3.0 mm posterior and 2.2 mm lateral to bregma). After 5 days recovery from surgery, mice were habituated for 2–3 days to the headstage (RHD2132 16-channel amplifier/accelerometer board, Part # C3335, Intan) and cable (RHD2000 6-ft Ultra-Thin SPI cable, Part # C3216, Intan) that are connected to the electrode on their heads. To ensure that the animal can move freely, the cable was suspended by a helium balloon. Electrical signals were continuously recorded in freely moving mice at a sampling rate of 2 KHz and recorded with Intan 512ch Recording Controller (Part #C3004, Intan). Animal behaviors were monitored with a digital camera mounted above (100 cm) the apparatus. Offline sorting software v4.4.1 and Neuroexplorer v5.0 (Plexon, Dallas, TX) were used to assess the power spectrum of the EEG signal (filtered 0.1–250 Hz). EEG power was calculated at five different frequency bands: delta (0.1–4 Hz), theta (4–8 Hz), alpha (8–13 Hz), beta (13–30 Hz), and gamma (30–60 Hz). For the rescue experiment, EEG was recorded at 1–3 days after the administration of CNO plus diazepam or vehicle (twice daily for 3 days).

### Behavioral testing

All behavioral tests were carried out at 2–3 weeks after treatment.

#### Social preference test

A three-chamber social interaction assay was performed to assess social deficits^36,67,69^. Briefly, an apparatus (L: 101.6 cm, W: 50.8 cm, H: 50.8 cm) containing three chambers with retractable doorways allowing for access to side chambers was used. Animals were habituated in the apparatus for one day before testing. During the habituation, two empty capsules (inverted pencil cup, D: 10.2 cm, H: 10.5 cm) were placed at the corners of chambers, and an upright cup was placed on top of each capsule to prevent the subject mouse from climbing on top. Animals were allowed to explore all 3 chambers of the apparatus for 10 m. The test was composed of two phases with different stimulus in each of side chambers. The 1^st^ phase contained two identical nonsocial stimuli (folded papers), the 2^nd^ phase contained a nonsocial (NS) stimulus (a woodblock) and a social (Soc) stimulus (an age- and sex-matched wild-type mouse of the same strain). Each stimulus was placed inside a capsule placed at the corner of each side chamber. The test animal was placed in the center chamber, and was free to explore the apparatus for 10 m in each phase, while it was returned to the home cage during the 10-min intervals between phases. The chamber was cleaned with 75% Ethanol after each phase. Interaction time was counted based on “investigating” behaviors of the test animal to each stimulus. A computer running the Any-maze tracking software (Stoelting, Wood Dale, IL) measured the time of the test animal spent at the close proximity of the capsule (distance of animal head to cup edge: ≤3.5 cm). Preference index scores were calculated, where time spent with one stimulus was subtracted from the time spent with the other stimulus and divided by the total time spent exploring both stimuli.

#### Self-grooming

Mice were scored for spontaneous grooming behaviors when placed individually in a clean cage. The cage was lined with a thin layer of bedding (~1 cm) in order to reduce neophobia but prevent digging, a potentially competing behavior. Prior to the testing period, animals were allowed to habituate to the novel environment for 10 min. Each mouse was rated for 10 min on cumulative time spent grooming.

#### Open field test

Animals were placed on an apparatus (L: 67.7 cm, W: 50.8 cm, H: 50.8 cm) to move freely for 10 m. The total distance traveled and the amount of time the animal spent in the center (33.8 cm × 25.4 cm) was counted by Any-maze tracking software (Stoelting, Wood Dale, IL). Anxious animals spend less time in the center and more time in the corner of the field.

### Statistical analysis

Data were analyzed with GraphPad Prism 7 (GraphPad), Clampfit (Molecular Devices, Sunnyvale, CA), and Mini analysis (Synaptosoft, NJ). All values are means ± SEM. Differences between two groups were assessed with unpaired two-tailed Student’s *t* test. Differences between more than two groups were assessed with one-way or two-way ANOVA, followed by *post hoc* Bonferroni tests for multiple comparisons.

### Reporting Summary

Further information on research design is available in the Nature Research Reporting Summary linked to this article.

## Supplementary information

Supplementary Information

Reporting Summary

Peer Review File

Supplementary Data 1

Supplementary Data 2

Supplementary Data 3

Supplementary Data 4

Supplementary Data 5

Supplementary Data 6

Supplementary Data 7

Description of Additional Supplementary Files

## Data Availability

The RNAseq data generated in this study have been deposited in the GEO public repository under accession code GSE181819. Source data are provided with this paper.

## Source data

Source Data
