# Supplementary material for: Deficiency of autism risk factor ASH1L in prefrontal cortex induces epigenetic aberrations and seizures
Source: Nat Commun. 2021 Nov 15;12:6589. doi: 10.1038/s41467-021-26972-8 (PMC8593046; doi:10.1038/s41467-021-26972-8)
Supplement: Supplementary file 1 — Supplementary Information [file 41467_2021_26972_MOESM1_ESM.pdf]

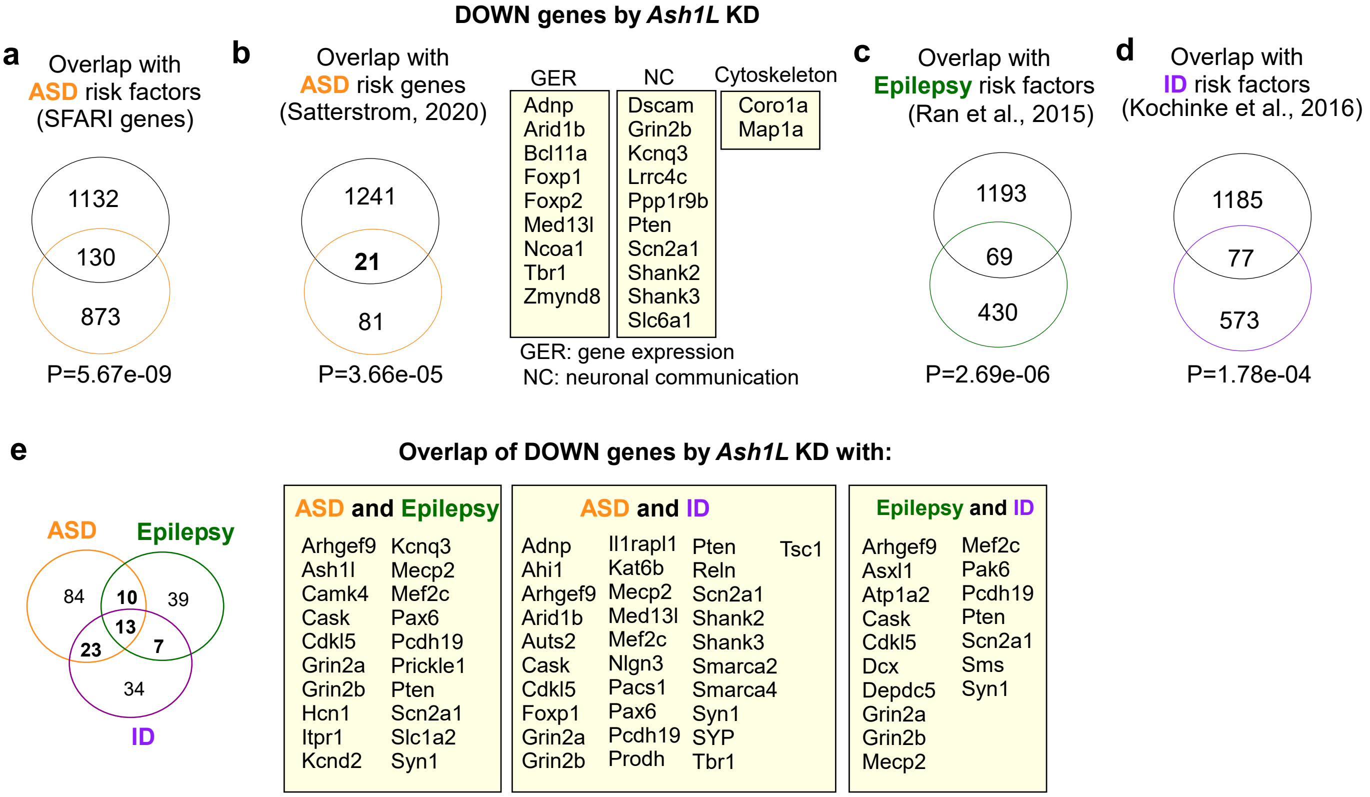

Sup. Figure 1. Genes downregulated by *Ash1L* deficiency in PFC overlap with risk factors for ASD, epilepsy and ID. (a-d) Venn diagram showing the overlapping of downregulated genes by *Ash1L* deficiency with the SFARI ASD risk genes (a), 102 high-risk ASD genes (b), epilepsy risk genes (c), or ID risk genes (d). Inset (b): List of overlapped ASD genes involved in 3 categories - Gene Expression Regulation (GER), Neuronal Communiation (NC), and Cytoskeleton. **(e)** Venn diagram and gene list showing the overlapping of downregulated genes by *Ash1L* deficiency with ASD, epilepsy and ID risk genes. **Related to Fig. 2.**

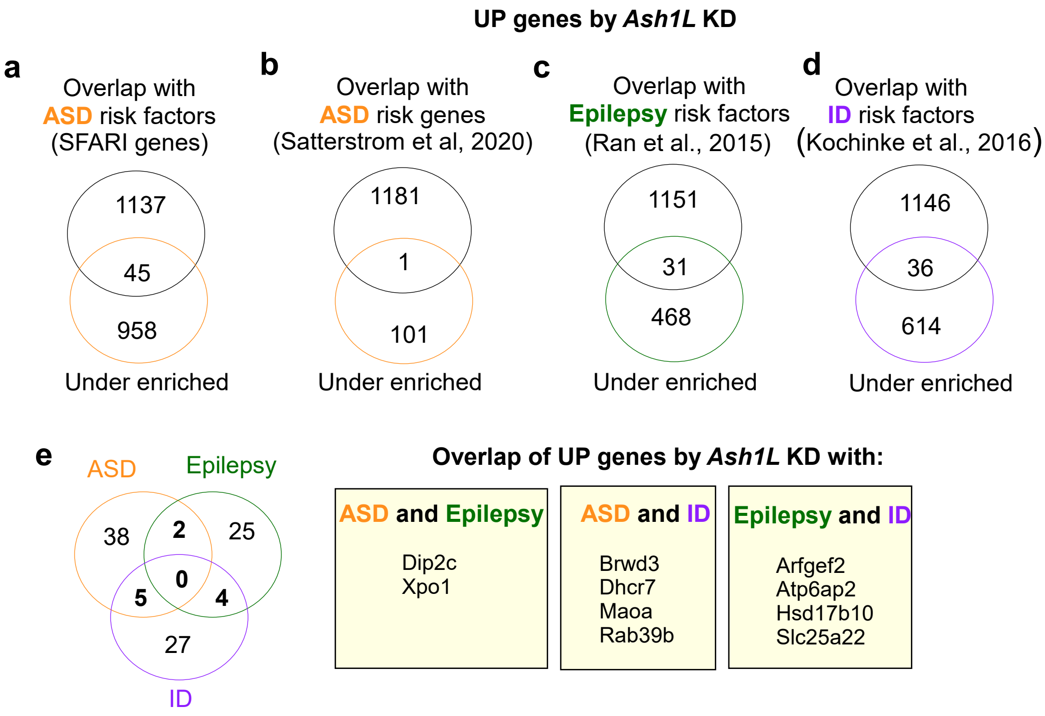

Sup. Figure 2. Genes upregulated by *Ash1L* deficiency in PFC do not significantly overlap with risk factors for ASD, epilepsy and ID. (a-d) Venn diagram showing the overlapping of upregulated genes by *Ash1L* deficiency with the SFARI ASD risk genes (a), 102 high-risk ASD genes (b), epilepsy risk genes (c), or ID risk genes (d). **(e)** Venn diagram and gene list showing the overlapping of upregulated genes by *Ash1L* deficiency with ASD, epilepsy and ID risk genes. **Related to Fig. 2.**

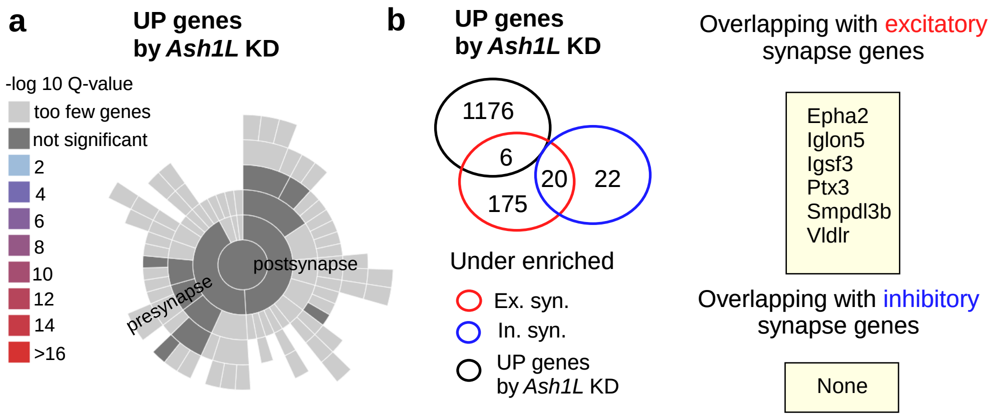

Sup. Figure 3. Genes upregulated by *Ash1L* deficiency in PFC are not enriched in the regulation of synaptic homeostasis. (a) Sunburst plot representing cellular component enrichment analysis of upregulated synaptic genes by *Ash1L* deficiency. No significant enrichment of upregulated genes was found in SynGO database. **(b)** Venn diagram showing the overlapping of upregulated genes by *Ash1L* deficiency and genes at excitatory or inhibitory synapses. Inset: the list of overlapped synaptic genes. **Related to Fig. 2.**

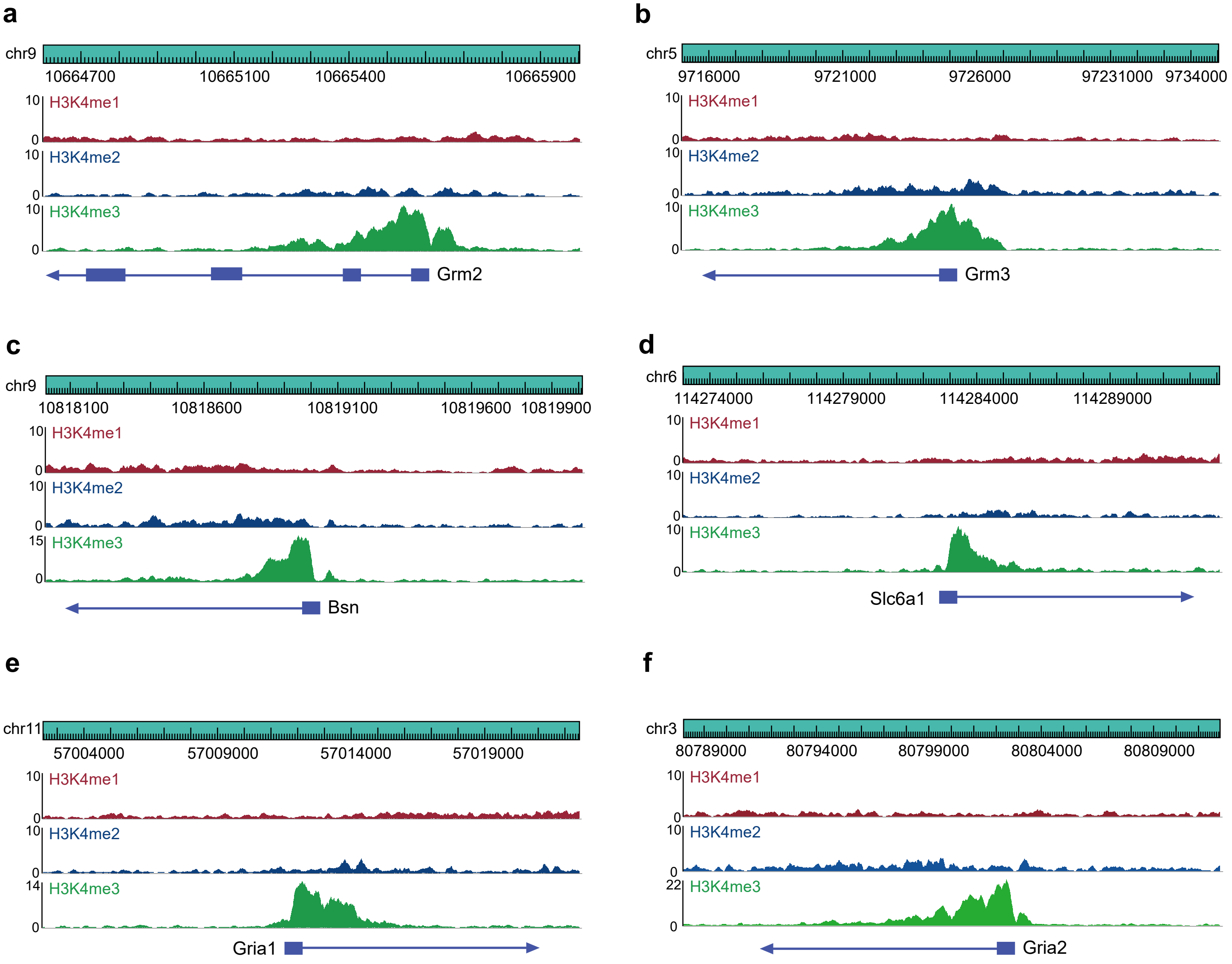

Sup. Figure 4. ChIPseq data showing the landscape of H3K4me1, H3K4me2, and H3K4me3 around the transcription start sites of several synaptic genes. (a-f) Genome browser snapshots of H3K4me1, H3K4me2, and H3K4me3 (GSM3508773, GSM3508775, GSM3508777) at promoter regions of *Grm2* (a), *Grm3* (b), *Bsn* (c), *Slc6a1* (d), *Gria1* (e) and *Gria2* (f). **Related to Fig. 3.**

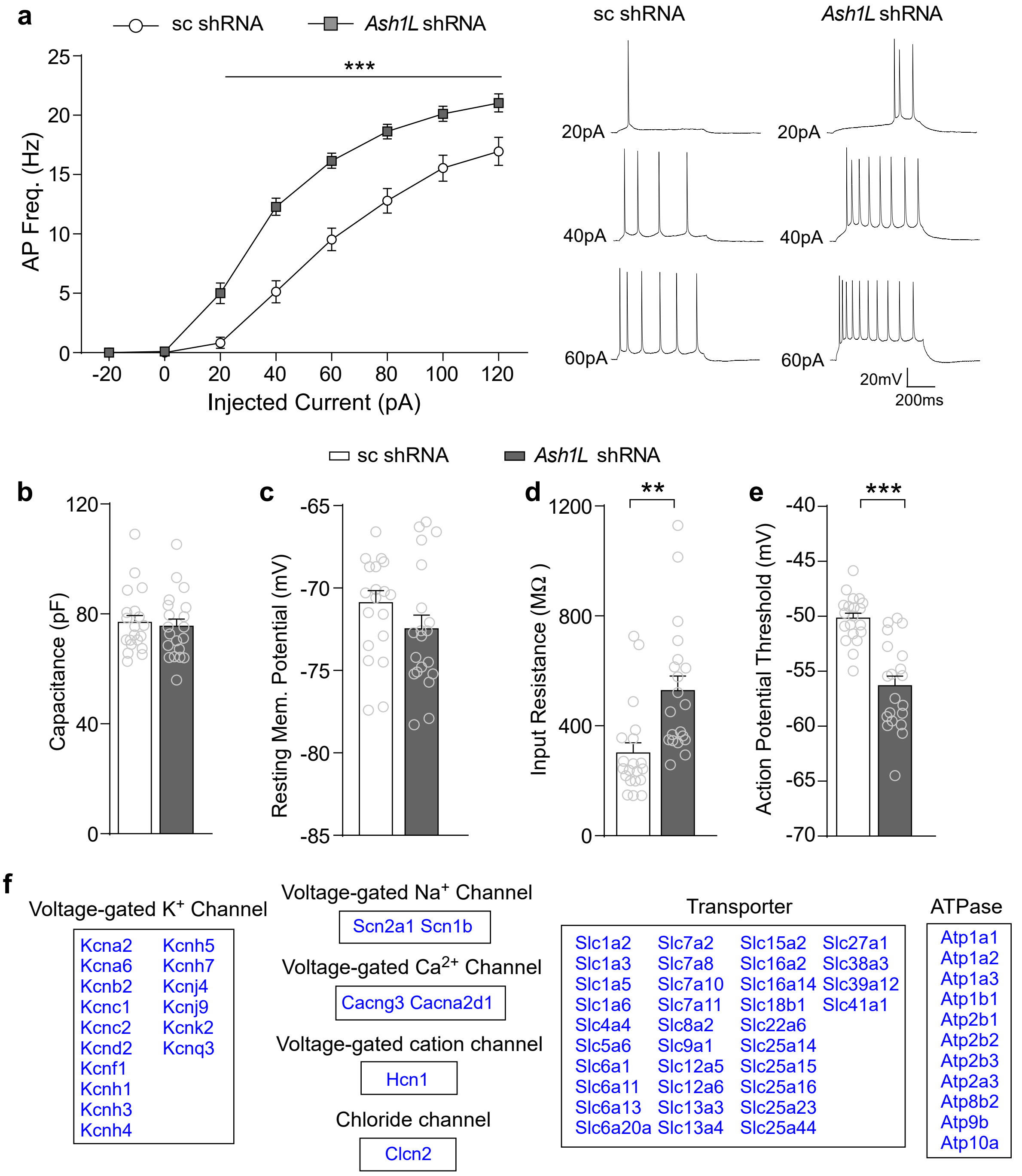

Sup. Figure 5. *Ash1L* deficiency increases intrinsic excitability, which is associated with the downregulation of ion channels and transporters. (a) Quantification of action potential (AP) frequencies in response to injected currents in PFC pyramidal neurons from mice infected with scrambled shRNA or *Ash1L* shRNA AAV. n=20 cells/4 mice(2M,2F)/group. ***p<0.001, two-way repeated ANOVA. Inset: representative AP traces. **(b-e)** Bar graphs showing the intrinsic properties of PFC pyramidal neurons from mice infected with scrambled shRNA or *Ash1L* shRNA AAV, including capacitance (b), resting membrane potential (c), input resistance (d) and action potential threshold (e). n=20 cells/4 mice(2M,2F)/group, **p<0.01, ***p<0.001, t-test. **(f)** List of genes encoding ion channels, transporters and transporting ATPases that are downregulated by *Ash1L* deficiency in PFC. Data are presented as mean values ± SEM. Detailed statistical data are provided in a Source Data file. Related to **Fig. 4.**

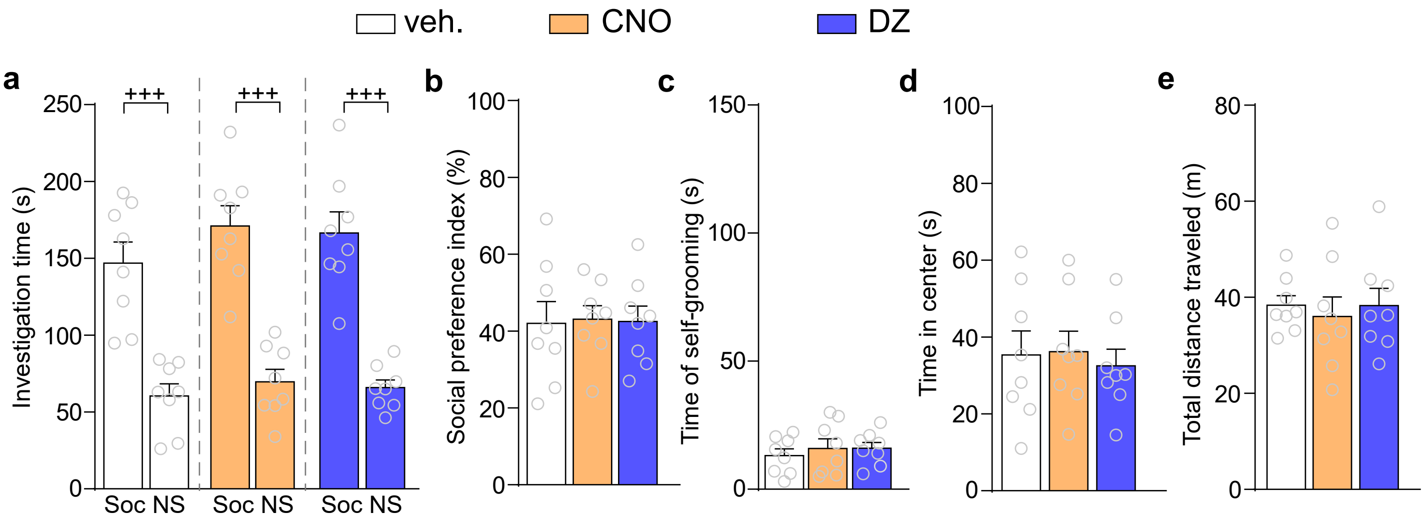

Sup. Figure 6. Clozapine-N-oxide (CNO) or diazepam (DZ) alone does not alter behaviors. (a, b) Bar graphs showing the amount of time spent on interacting with the social (Soc) vs. non-social (NS) stimulus (a) and social preference index (b) in 3-chamber social preference tests of control mice (infected with scrambled shRNA AAV) treated with CNO or DZ (n=8/group). +++p<0.001, two-way ANOVA. **(c-e)** Bar graphs showing the time spent in self-grooming (c), the time in center of open field tests (d) and the distance traveled in locomotion tests (e) of CNO- or DZ-treated mice (n=8/group). Data are presented as mean values ± SEM. Detailed statistical data are provided in a Source Data file. Related to **Fig. 6.**

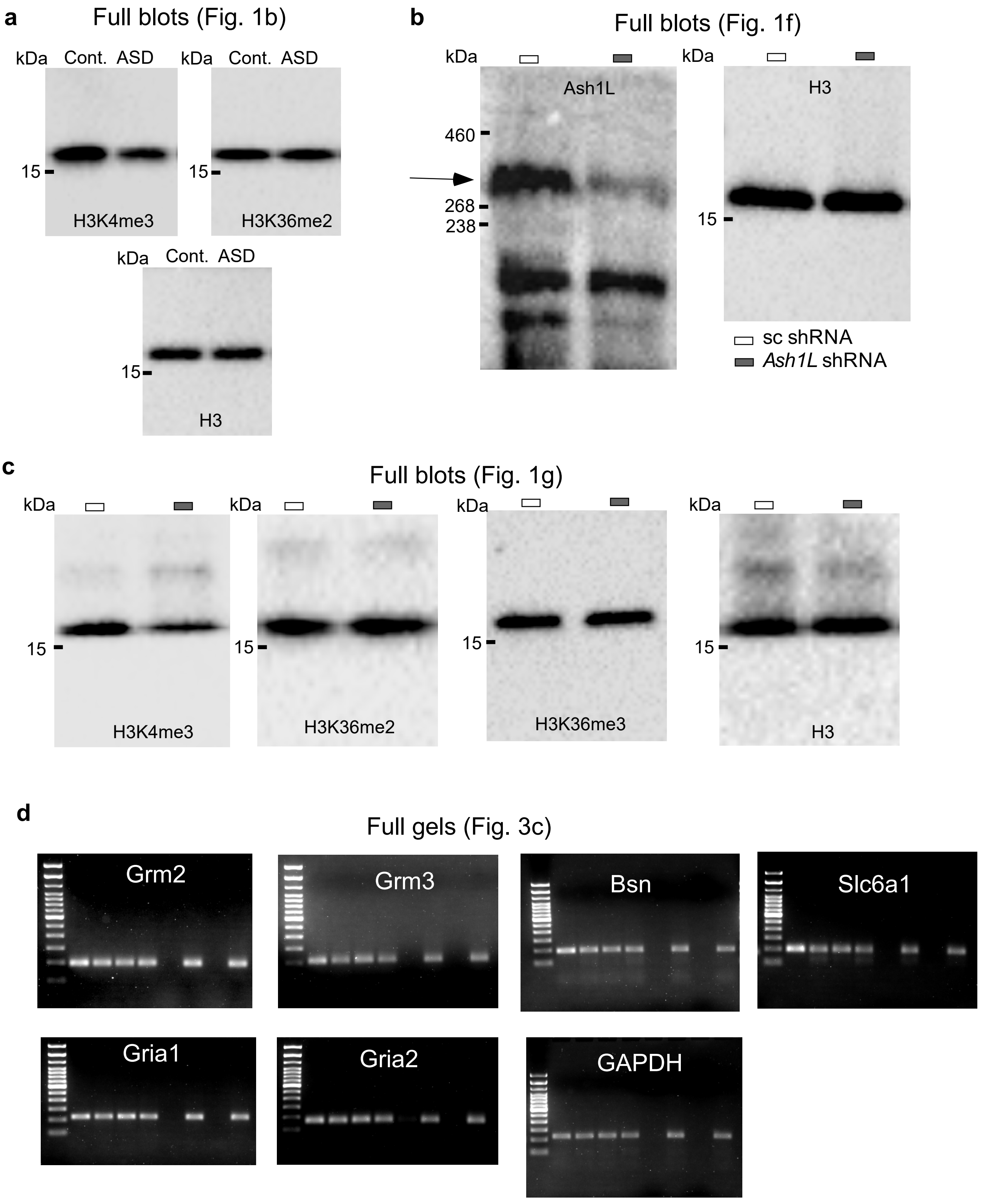

Sup. Figure 7. Full Western blots and PCR gels. Related to **Fig. 1** and **Fig. 3**.
