# Supplementary material for: Deficiency of autism risk factor ASH1L in prefrontal cortex induces epigenetic aberrations and seizures
Source: Nat Commun. 2021 Nov 15;12:6589. doi: 10.1038/s41467-021-26972-8 (PMC8593046; doi:10.1038/s41467-021-26972-8)
Supplement: Supplementary file 2 — Reporting Summary [file 41467_2021_26972_MOESM2_ESM.pdf]

nature portfolio

Corresponding author(s): Zhen Yan
Last updated by author(s): Oct 15, 2021

# Reporting Summary

Nature Portfolio wishes to improve the reproducibility of the work that we publish. This form provides structure for consistency and transparency in reporting. For further information on Nature Portfolio policies, see our Editorial Policies and the Editorial Policy Checklist.

## Statistics

For all statistical analyses, confirm that the following items are present in the figure legend, table legend, main text, or Methods section.

| n/a | Confirmed | |
|---|---|---|
| ☐ | ☒ | The exact sample size (*n*) for each experimental group/condition, given as a discrete number and unit of measurement |
| ☐ | ☒ | A statement on whether measurements were taken from distinct samples or whether the same sample was measured repeatedly |
| ☐ | ☒ | The statistical test(s) used AND whether they are one- or two-sided<br>*Only common tests should be described solely by name; describe more complex techniques in the Methods section.* |
| ☐ | ☒ | A description of all covariates tested |
| ☐ | ☒ | A description of any assumptions or corrections, such as tests of normality and adjustment for multiple comparisons |
| ☐ | ☒ | A full description of the statistical parameters including central tendency (e.g. means) or other basic estimates (e.g. regression coefficient) AND variation (e.g. standard deviation) or associated estimates of uncertainty (e.g. confidence intervals) |
| ☐ | ☒ | For null hypothesis testing, the test statistic (e.g. *F*, *t*, *r*) with confidence intervals, effect sizes, degrees of freedom and *P* value noted<br>*Give P values as exact values whenever suitable.* |
| ☒ | ☐ | For Bayesian analysis, information on the choice of priors and Markov chain Monte Carlo settings |
| ☐ | ☒ | For hierarchical and complex designs, identification of the appropriate level for tests and full reporting of outcomes |
| ☐ | ☒ | Estimates of effect sizes (e.g. Cohen's *d*, Pearson's *r*), indicating how they were calculated |

*Our web collection on statistics for biologists contains articles on many of the points above.*

## Software and code

Policy information about availability of computer code

| | |
|---|---|
| Data collection | Behavioral data were acquired with a computer running the Any-maze tracking software.<br><br>Electrophysiological data were collected by Clampex software (MDS Analytical Technologies, Sunnyvale, CA).<br><br>EEG data were recorded by using RHD recording system (Intan Technologies, USA).<br><br>Images were acquired by a Leica TCS SP8 confocal microscope (Leica Microsystems).<br><br>Quantitative real time PCR data were collected by iCycler iQ™ RealTime PCR Detection System.<br><br>RNA sequencing was carried out with the HiSeq 2500 platform (Illumina) at the Genomics and Bioinformatics Core of the State University of New York at Buffalo. |
| Data analysis | Data analyses were performed with Clampfit 9, Mini Analysis and GraphPad Prism 7. EEG data were analyzed by offline sorter v4.4.1 and NeuroExplorer® v5.0 (Plexon, Dallas, TX).<br><br>Experiments with two groups were analyzed statistically using two-tailed Student's t-tests. Experiments with more than two groups were subjected to one-way ANOVA, two-way ANOVA, or two-way repeated measure ANOVA (rmANOVA), followed by post hoc Bonferroni tests. We described this in the Method section of the manuscript.<br><br>RNA sequencing date analyses were performed by using RNA STAR (Galaxy version 2.7.5b), featureCounts (Galaxy Version 1.6.4+galaxy1) and |

DEseq2 (Galaxy Version 2.11.40.2).

For manuscripts utilizing custom algorithms or software that are central to the research but not yet described in published literature, software must be made available to editors and reviewers. We strongly encourage code deposition in a community repository (e.g. GitHub). See the Nature Portfolio guidelines for submitting code & software for further information.

## Data

Policy information about availability of data

All manuscripts must include a data availability statement. This statement should provide the following information, where applicable:

- Accession codes, unique identifiers, or web links for publicly available datasets
- A description of any restrictions on data availability
- For clinical datasets or third party data, please ensure that the statement adheres to our policy

The RNAseq data generated in this study have been deposited in the GEO public repository under accession code GSE181819 (https://www.ncbi.nlm.nih.gov/geo/query/acc.cgi?acc=GSM5512486). Source data are provided with this paper.

# Field-specific reporting

Please select the one below that is the best fit for your research. If you are not sure, read the appropriate sections before making your selection.

[x] Life sciences [ ] Behavioural & social sciences [ ] Ecological, evolutionary & environmental sciences

For a reference copy of the document with all sections, see nature.com/documents/nr-reporting-summary-flat.pdf

# Life sciences study design

All studies must disclose on these points even when the disclosure is negative.

| | |
|---|---|
| Sample size | Sample sizes were based on power analyses and previous experiments conducted by us (Yuen EY et al., Neuron 73:962-77, 2012; Duffney LJ et al., Cell Reports 11:1400-1413, 2015; Wei J et al., J. Neuroscience 36:2119-30, 2016) and many other labs in the field. |
| Data exclusions | No data was excluded in analysis. |
| Replication | We listed the number of times we repeated the experiments in figure legends. The behavioral phenotypes reported here (by L. Qin) were independently replicated by 3 other lab members. |
| Randomization | Mice were randomly assigned to Ash1l shRNA/scrambled shRNA or drug/saline groups. |
| Blinding | The investigator was blinded to the group allocation (with no prior knowledge about Ash1l shRNA/scrambled shRNA injection or the treatments) during the experiments. |

# Reporting for specific materials, systems and methods

We require information from authors about some types of materials, experimental systems and methods used in many studies. Here, indicate whether each material, system or method listed is relevant to your study. If you are not sure if a list item applies to your research, read the appropriate section before selecting a response.

## Materials & experimental systems

| n/a | Involved in the study | |
|---|---|---|
| [ ] | [x] | Antibodies |
| [x] | [ ] | Eukaryotic cell lines |
| [x] | [ ] | Palaeontology and archaeology |
| [ ] | [x] | Animals and other organisms |
| [x] | [ ] | Human research participants |
| [x] | [ ] | Clinical data |
| [x] | [ ] | Dual use research of concern |

## Methods

| n/a | Involved in the study | |
|---|---|---|
| [x] | [ ] | ChIP-seq |
| [x] | [ ] | Flow cytometry |
| [x] | [ ] | MRI-based neuroimaging |

## Antibodies

Antibodies used

Antibody information is included in the Method section.

Western blotting: H3K4me3 (1:1000, Cell Signaling, 9751), H3 (1:500, Cell Signaling Technology, 4499), H3K36me2 (1:1000, Cell

| | |
|---|---|
| | Signaling Technology, 2901), H3K36me3 (1:1000, Cell Signaling, 9763), Ash1L (1:1000, LSBio, LS-B11718)<br><br>ChIP: H3K4me3 (Abcam, ab8580, 8 µl per reaction)<br><br>Immunohistochemistry: H3K4me3 (1:1000, Cell Signaling, 9751), NeuN (1:1000, Millipore, MAB377), Alexa Fluor 594 (1:1000, Invitrogen, A11037) |
| Validation | All antibodies have been validated by vendors or by our current and previously published studies. |

## Animals and other organisms

Policy information about studies involving animals; ARRIVE guidelines recommended for reporting animal research

| | |
|---|---|
| Laboratory animals | Wild-type (WT) mice (5 weeks old, male and female) with C57BL/6J background were used in this study. |
| Wild animals | This study did not involve wild animals. |
| Field-collected samples | The study did not involve samples collected from the field. |
| Ethics oversight | All experiments were performed with the approval of the Institutional Animal Care and Use Committee (IACUC) of the State University of New York at Buffalo. |

Note that full information on the approval of the study protocol must also be provided in the manuscript.
