# Supplementary material for: Deficiency of autism risk factor ASH1L in prefrontal cortex induces epigenetic aberrations and seizures
Source: Nat Commun. 2021 Nov 15;12:6589. doi: 10.1038/s41467-021-26972-8 (PMC8593046; doi:10.1038/s41467-021-26972-8)
Supplement: Supplementary file 11 — Description of Additional Supplementary Files [file 41467_2021_26972_MOESM11_ESM.pdf]

**Title: Supplementary Data 1.**
Downregulated genes by ASH1L knockdown in PFC

**Title: Supplementary Data 2.**
Upregulated genes by ASH1L knockdown in PFC

**Title: Supplementary Data 3.**
Overlapping of downregulated genes by ASH1L knockdown in PFC with ASD/Epilepsy/ID risk factors

**Title: Supplementary Data 4.**
GO analysis of downregulated genes by ASH1L knockdown in PFC

**Title: Supplementary Data 5.**
GO analysis of upregulated genes by ASH1L knockdown in PFC

**Title: Supplementary Data 6.**
Information about human postmortem tissues used in this study

**Title: Supplementary Data 7.**
List of primers used in qPCR experiments
